# Transcriptome profiling in Rift Valley fever virus infected cells reveals modified transcriptional and alternative splicing programs

**DOI:** 10.1371/journal.pone.0217497

**Published:** 2019-05-28

**Authors:** Katherine E. Havranek, Luke Adam White, Jean-Marc Lanchy, J. Stephen Lodmell

**Affiliations:** 1 Division of Biological Sciences, The University of Montana, Missoula, MT, United States of America; 2 Center for Biomolecular Structure and Dynamics, The University of Montana, Missoula, MT, United States of America; University of Texas Medical Branch at Galveston, UNITED STATES

## Abstract

Rift Valley fever virus (RVFV) is a negative-sense RNA virus belonging to the *Phenuiviridae* family that infects both domestic livestock and humans. The NIAID has designated RVFV as a Category A priority emerging pathogen due to the devastating public health outcomes associated with epidemic outbreaks. However, there is no licensed treatment or vaccine approved for human use. Therefore it is of great interest to understand RVFV pathogenesis in infected hosts in order to facilitate creation of targeted therapies and treatment options. Here we provide insight into the host-pathogen interface in human HEK293 cells during RVFV MP-12 strain infection using high-throughput mRNA sequencing technology. Gene ontology (GO) and Kyoto Encyclopedia of Genes and Genomes (KEGG) analysis of differentially expressed genes showed robust innate immune and cytokine-mediated inflammatory pathway activation as well as alterations in pathways associated with fatty acid metabolism and extracellular matrix receptor signaling. We also analyzed the promoter regions of DEGs for patterns in transcription factor binding sites, and found several that are known to act synergistically to impact apoptosis, immunity, metabolism, and cell growth and differentiation. Lastly, we noted dramatic changes in host alternative splicing patterns in genes associated with mRNA decay and surveillance, RNA transport, and DNA repair. This study has improved our understanding of RVFV pathogenesis and has provided novel insight into pathways and signaling modules important for RVFV diagnostics and therapeutic development.

## Introduction

Rift Valley fever virus (RVFV) is a mosquito-borne zoonotic disease that was originally discovered in the Kenyan Rift basin in the 1930s [1]. The virus has since spread throughout Sub-Saharan Africa and has more recently extended its reach outside of the African continent into the Arabian Peninsula [2]. More than fifty mosquito vector species can transmit the virus, including species whose home range spans well into portions of the Americas, such as *Aedes aegypti* mosquitos that carry other viruses like Zika, chikungunya, yellow fever and dengue [3]. RVFV causes severe disease in both livestock and humans. Epidemics in livestock yield 10–20% mortality in adults and cause what have been termed ‘abortion storms’ in fetuses and neonates, since mortality rates can rise as high as 100% [4]. In humans, the virus causes an array of clinical manifestations ranging from mild flu-like illness to severe complications including blindness, meningoencephalitis, hemorrhagic fever and death [5]. High rates of human infection coincide with above average periods of rainfall and flooding, which provide ripe conditions for an overabundance of mosquito vectors. RVFV has already demonstrated both its potential for spread outside of endemic regions and its ability to cause devastating public health and economic impacts. Furthermore, there is no licensed vaccine for human use, although there is ongoing vaccine testing in livestock [6].

RVFV is a tri-segmented negative-sense single-stranded RNA virus belonging to the *Phlebovirus* genus within the *Phenuiviridae* family [7]. The viral genome consists of three segments designated large (L), medium (M) and small (S), which encode the viral RNA-dependent RNA polymerase, the viral glycoproteins Gn and Gc and nonstructural protein NSm, and the viral nucleocapsid protein N and the nonstructural virulence protein, NSs, respectively. These few viral proteins, in conjunction with host proteins, ensure that the virus can replicate its genome during a productive infection. Virus-host interaction is paramount for viral progeny production and therefore it is of great interest to understand how these interactions shape the cellular landscape during viral infection. The rising trend of high throughput mRNA sequencing has enabled a profound appreciation for the cellular transcriptomic overhaul that occurs as a result of disease progression, cancer state, or environmental condition [8–13]. However, relatively few mRNA-seq studies to date have characterized the host response to viral infection. The studies that have risen to this challenge have revealed novel insights into host-pathogen dynamics [14–18]. A deeper understanding of the host response to viral infection will contribute to the development of effective treatment options and targeted therapies. In this study our objective was to characterize changes in gene expression that occur during RVFV infection, as well as alterations in transcription factor usage and host splicing.

## Materials and methods

### Cell culture and viral infections

HEK293 cells were cultured in Dulbecco’s Modified Eagle Medium (DMEM) with 10% fetal bovine serum and 1% penicillin/streptomycin. For experiments using MP-12 RVFV, 80–90% confluent HEK293 cells were washed with phosphate-buffered saline (PBS) and overlaid with virus at the specified MOI in DMEM without serum or antibiotics and incubated for one hour at 37°C and 5% CO_2_. After incubation with virus the media was replaced with DMEM supplemented with 2% fetal bovine serum and the cells were maintained at 37°C and 5% CO_2_ until time of harvest. Viral infection was confirmed using immunofluorescence targeting the viral nucleocapsid protein as well as RT-qPCR amplification of the viral small genome segment. The MP-12 vaccine strain of RVFV was kindly provided by Brian Gowen (Utah State University, Logan, UT). The virus stock was amplified once on Vero cells and portioned into small aliquots and frozen so that all experiments used the same passage of virus.

### RNA Sequencing

Total RNA was extracted from infected and uninfected cells using the PureLink RNA Mini Kit (Thermo Fisher Scientific) and RNA integrity was verified using TapeStation 2200 Bioanalyzer (Agilent) and agarose gel electrophoresis. High quality RNA samples representing triplicate infected or mock-infected cells were submitted to Novogene for commercial RNA-sequencing (Novogene Beijing, China). Poly-A mRNA was selected from total RNA using oligo-dT bound magnetic beads and strand specific NEBNext Ultra RNA libraries were synthesized for paired-end 150 bp (PE 150) on an Illumina HiSeq 2000 platform (Illumina Inc.). Base calling was performed using CASAVA and raw data was stored as FASTQ files. Reads were filtered for quality and aligned to the GRCh-37 (hg19) genome using TopHat2 to generate BAM files [19]. DESeq2 R package was used to identify differentially expressed genes (DEGs) in MP-12 infected cells relative to mock infected cells [20]. For further differential expression analysis, we employed a threshold of p<0.05 and log2>1.8 (3.5 fold change). Since even these stringent parameters yielded a large number of DEGs, a ranked approach using lists organized by log2 fold change for up- and down- regulated genes was input for GOrilla and then REVIGO, which performed gene ontology analysis of differentially expressed genes and removal of redundant GO terms, respectively [21, 22]. KEGG pathway analysis of DEGs was analyzed by the Database for Annotation, Visualization and Integrated Discovery (DAVID) [23]. oPOSSUM3 was used in single site analysis (SSA) mode to detect conserved transcription factor binding sites in significantly differentially expressed genes [24]. The oPOSSUM3 default setting for gene-based analyses was used, in which all genes in the oPOSSUM database serve as background. The Mixture of Isoforms (MISO) framework was used for identification of alternatively spliced genes [25]. KEGG pathway analysis of differentially spliced genes was also carried out using DAVID.

### Flow Cytometry

To establish infection and live/dead counts for cells harvested for RNA sequencing analysis, another set of HEK293 cells was either mock-infected or infected with RVFV MP-12 strain at an MOI = 0.1 and harvested at 48hpi with trypsin/EDTA. After quenching the trypsin with serum-containing media, cells were pelleted by centrifugation and resuspended in PBS (1 x 10^6^ cells per tube; 1 ml PBS). Cells were then incubated in the dark with a live/dead cells differential staining kit according to the manufacturer's instructions (Thermo LIVE/DEAD Fixable Red Dead Cell Stain Kit). All incubations used to prepare samples for flow cytometry were carried out at room temperature in the dark with gentle agitation. After two PBS washes, cells were fixed with fixation buffer for 30 min according to the manufacturer's instructions (Thermo eBioscience intracellular fixation & permeabilization buffer set). The fixed cells were permeabilized with two washes with permeabilization buffer. Cells were then incubated for 60 min with mouse monoclonal antibody directed against RVFV Gn protein (1:200 dilution in permeabilization buffer; BEI Resources). After two washes with permeabilization buffer, the cells were incubated for 30 min with a 1:200 dilution of goat anti-mouse green Alexa-Fluor 488-conjugated secondary antibody (Thermo). Finally, after two washes with permeabilization buffer, the cells pellets were resuspended in 1 ml PBS and stored at 4°C in the dark until flow cytometry analysis. Cells were analyzed on an LSR II (BD Biosciences) in the University of Montana Center for Translational Medicine. Data were analyzed with FlowJo 10 software (Tree Star, Ashland, OR).

### Data

Sequence data analyses are supplied as supplemental information with this manuscript.

### RT-qPCR

Total RNA was extracted using the PureLink RNA Mini Kit (Thermo Fisher Scientific) and 600ng of RNA was reverse transcribed using Superscript III First-Strand Synthesis SuperMix (Thermo Fisher Scientific) with random hexamers or gene specific primers according to the manufacturer’s instructions. qPCR was performed using the Applied Biosystems Step One Real-Time PCR System. For relative RT-qPCR, RNA levels were normalized to GAPDH and fold change in expression was calculated using the ΔΔCT method [26]. The effect of MP-12 infection on GAPDH expression is displayed as a supplementary figure to validate use of GAPDH as an internal control gene (S2 Fig). Quantitative RT-qPCR of the small genome segment was carried out as previously described and standard curves were generated using in vitro synthesized RNA [27]. Primers used in this study are as follows listed 5’ to 3’: GAPDH (Qiagen Quantitect Primer Assay), IFNB: F- AAACTCATGAGCAGTCTGCA, R- AGGAGATCTTCAGTTTCGGAGG, CXCL10: F- TGGCATTCAAGGAGTACCTCTCT, R- CTGATGCAGGTACAGCGTACG

IFIT2: F- TGCACTGCAACCATGAGTGAGAACA, R- GCCAGTAGGTTGCACATTGTGGC, mir210hg: F- GGCAGATTTAGTGGACGCCT, R- CTCACTTCGCAGTGGTGACA, NOG: F- GTGCAAGTGCTCGTGCTAGA, R- GCTAGAGGGTGGTGGAACTG

### RT-PCR

Validation of alternative splicing events via RT-PCR Primers were designed using Primer-BLAST [28], listed here (5’ to 3’): F_RIOK3_Ex5 CCGGTTCCCACTCCTAAAAAGGGC; R_RIOK3_Ex10 CCAGCATGCCACAGCATGTTATACTCAC; F_TRA2B_Ex1 AGGAAGGTGCAAGAGGTTGG; R_TRA2B_Ex3 TCCGTGAGCACTTCCACTTC; F_CLK2_Ex3 CCGGACATTTAGCCGCTCAT; R_CLK2_Ex6 TGGCCATGGTAGTCAAACCA; F_DDX5_Ex11 ATTGCTACAGATGTGGCCTCC; R_DDX5_Ex12 TGCCTGTTTTGGTACTGCGA; F_18S_rRNA GTAACCCGTTGAACCCCATT; R_18S_rRNA CCATCCAATCGGTAGTAGCG. Cells were infected or mock infected and harvested after 48 hours, and RNA was isolated via TRIzol. cDNA was generated using 0.1 μg total RNA, Maxima H Minus reverse transcriptase (ThermoFisher), and random hexamer primer. The PCR reaction was comprised of 10**μ**l Phusion Flash High-Fidelity PCR Master Mix (ThermoFisher), 1**μ**l each primer (10mM), 2**μ**l cDNA, and 7**μ**l H_2_O. Amplification was achieved with one cycle at 98°C for one minute, and 30 cycles of denaturation at 98°C for one second followed by paired annealing and extension at 72°C for 15 seconds. RT-PCR reactions were analyzed via agarose gel electrophoresis (S1 Fig).

## Results

### Transcriptome profiling and differential expression in RVFV MP-12 infected cells

**Fig 1:**
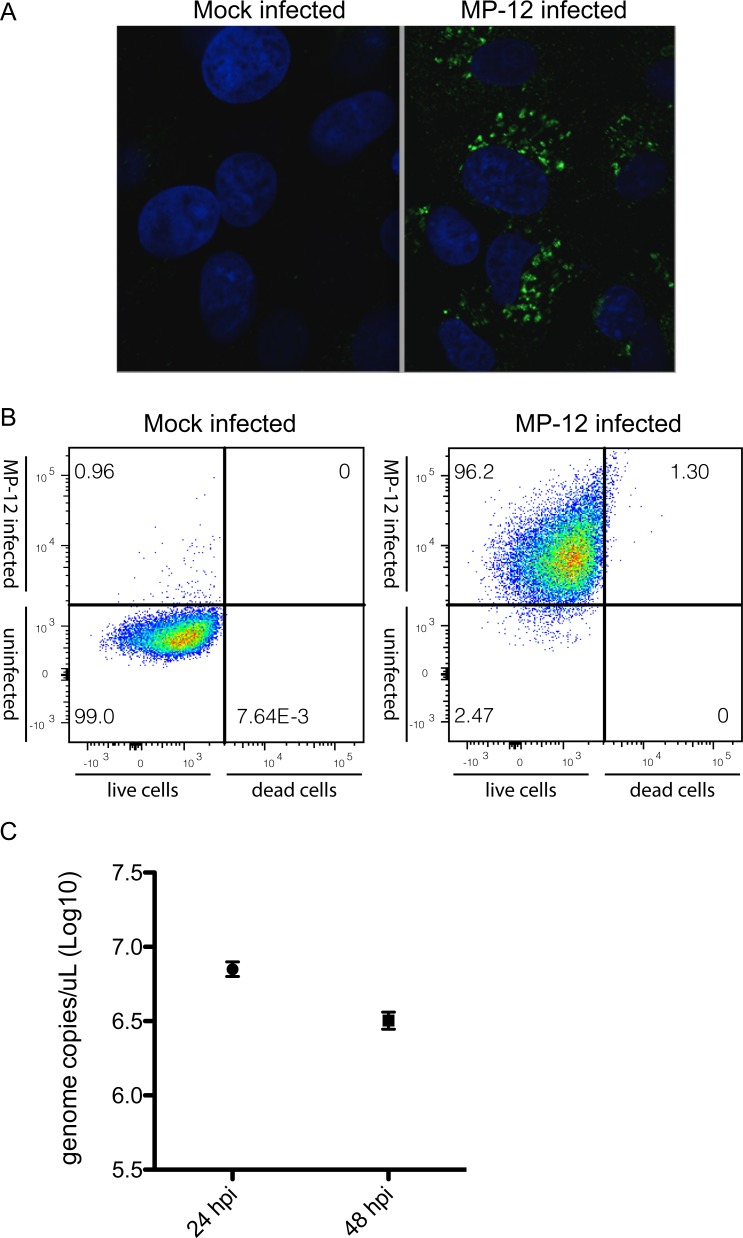
RVFV MP-12 infection in HEK293 cells at MOI = 0.1. A. Immunofluorescent detection of viral nucleocapsid protein in infected and mock-infected cells 48hpi. B. Flow cytometry analysis of the infection levels and live/dead cells ratio at 48hpi of mock-infected (left panel) and infected (right panel) cells. The X-axis represents the red fluorescence associated with the live/dead cell staining. Live cells are on the left, dead cells on the right of the vertical cut-off line. The Y-axis represents the green fluorescence associated with infection (*i*.*e*., presence of viral Gn protein). Infected cells are on the top, uninfected cells on the bottom of the horizontal cut-off line. The fraction (percent) of each of the four possible combinations (dead/live, uninfected/infected) is indicated in the corner of each quadrant. C. RT-qPCR absolute quantification of the viral sense small genome segment at timepoints post-infection. Each point represents the mean copy number during infections performed in triplicate.

In the present study, mRNA-seq was performed to compare transcriptomes in RVFV infected and uninfected HEK293 cells. Cells were either mock-infected or infected with RVFV MP-12 strain at an MOI = 0.1 and total RNA was harvested at 48hpi. RVFV MP-12 is an attenuated vaccine candidate strain that allows viral work to be completed under BSL2 containment conditions. Immunofluorescence, flow cytometry, and RT-qPCR confirmed that most cells were infected at the time of harvest using this scheme and that harvested cells were live (Fig 1).

Our goal in this study was to specifically analyze differential transcriptional and alternative splicing landscapes during RVFV infection. A related but distinct study detailing differential expression and pathway analysis at time points up to 18h post-RVFV infection in HSEAC cells was recently published [14]. Here we were interested in capturing a composite of statistically significant splicing events and changes in transcription factor usage occurring post-infection, therefore a synchronous infection was not ideal for our purposes. At 48 hours after infection at MOI = 0.1, immunofluorescence and flow cytometry indicated that nearly all cells were infected, and few were dead (Fig 1). This infection scheme allowed us to capture RNA from cells at different stages of infection, which was appropriate for observing highly significant and pervasive RNA processing events.

Poly-A selected RNA from three biological replicates was used for paired-end library preparation and transcriptome sequencing on an Illumina HiSeq platform. Approximately 136 million raw reads and 131 million clean reads were generated on average per sample, with phred Q30 values averaging 90.5% (S1 Table). Reads were mapped to the hg19 genome using TopHat2, which resulted in assembly of >85% of reads across samples [19] (S2 Table). RVFV mRNAs do not contain poly-A signals, therefore it was not necessary to map reads to the viral genome, since only polyadenylated RNAs were used for library generation.

**Fig 2:**
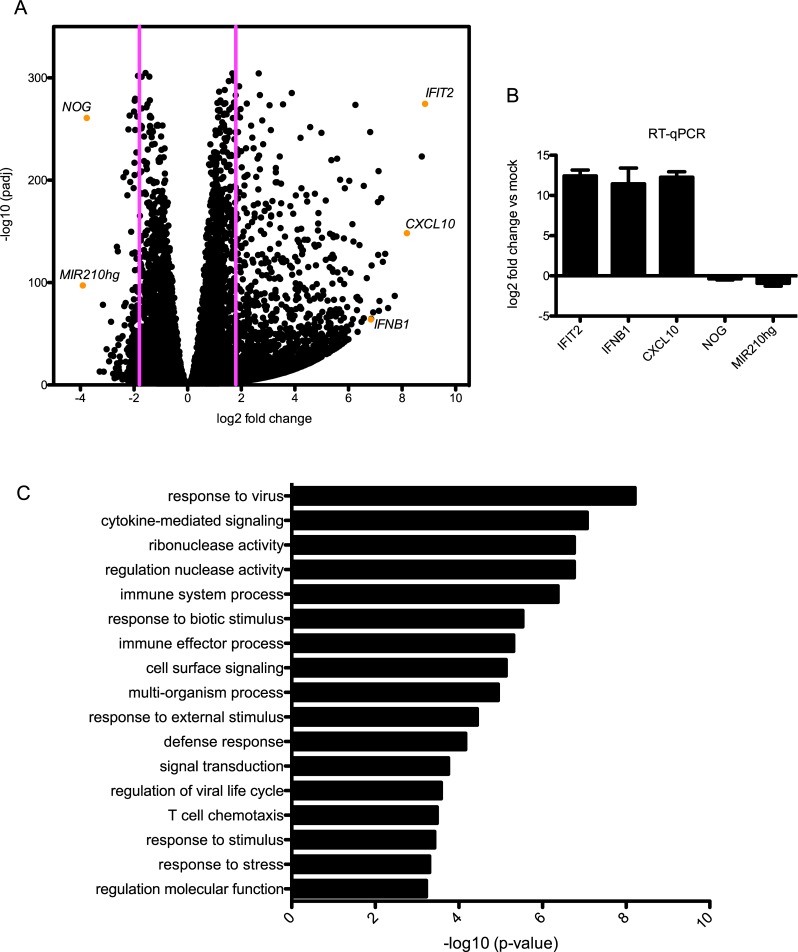
Differentially expressed genes and associated gene ontology terms at 48hpi. A. Volcano plot of DEGs illustrating significance cut-off set to log2 fold change of 1.8. DEGs validated by RT-qPCR are labeled. B. RT-qPCR confirmation of expression level changes in DEG analysis. C. GO terms associated with upregulated genes.

Differential expression comparison of infected and mock-infected cells was carried out using the DEGSeq2 R package [20] (S3, S4 and S5 Tables). A threshold of p<0.05 and log2>1.8 (3.5 fold change) yielded 3125 differentially expressed genes, 2909 upregulated (93%) and 216 (7%) downregulated genes (Fig 2A). Table 1 represents the top up- and down- regulated genes. The differential expression analysis was validated via RT-qPCR for 5 genes, 3 upregulated (CXCL10, IFIT2, IFNB) and 2 downregulated (MIR210hg, NOG) (Fig 2B). Gene expression during RVFV infection cannot be analyzed without taking into account the role of viral NSs protein. In infected cells, the 31kDa NSs is present in the cytoplasm as well as in the nucleus, where it forms filamentous structures mediated by the C-terminal domain [29]. NSs has been shown to suppress transcription on a global level through its interaction with transcription complex TFIIH components p44 and p62, which inhibits TFIIH complex assembly by sequestration of p44 and promotes posttranslational degradation of p62 [30, 31]. In addition to its role in transcription, TFIIH also plays roles in cell cycle control and DNA repair [32–34]. Despite these mechanisms, it is clear that there is an abundance of mRNA present at the 48h timepoint post-infection. In fact, the number of reads obtained from mock infected cells was only slightly higher than read count from MP-12 infected cells on average across the three replicates (S1 Table).

**Table 1:** Top up- and down- regulated genes sorted according to log2 fold change.

Top up-regulated genes		Top down-regulated genes	
Gene Symbol	Log 2 fold change	Gene Symbol	Log 2 fold change
IFIT2	8.8609	MIR210HG	-3.9125
OASL	8.7374	NOG	-3.7649
CXCL10	8.1793	OR51E2	-3.2826
IFI44	7.9412	RP6-24A23.7	-3.2239
OAS2	7.7257	RP11-214O1.2	-3.1586
OAS1	7.4817	ZNF346-IT1	-3.1292
MX2	7.3722	KIF12	-3.0213
C6orf222	7.2838	IRS4	-2.999
ITGAM	7.2273	C1orf95	-2.9308
FAP	7.1594	RP11-355I22.7	-2.8618
LHFPL3-AS1	7.1383	IGFBP5	-2.8572
CCL5	7.1212	RP11-250B2.3	-2.7806
SLC2A5	7.1094	KCNA2	-2.7728
IL16	7.0972	AC074212.5	-2.7277
PKHD1	6.9233	RP11-148O21.6	-2.7234
ANGPT2	6.8649	RP11-148O21.3	-2.6813
IFNB1	6.8447	POSTN	-2.6773
CPA2	6.8341	KDM5B-AS1	-2.6659
CGA	6.8093	HILPDA	-2.6617
IFIH1	6.7032	RP11-148O21.4	-2.6528

Upregulated genes are in good agreement with those from a transcriptome profiling study performed by Pinkham and colleagues with both MP-12 and ZH548 strains of RVFV over a shorter 18hr time-course of infection in HSEAC cells [14]. Their study showed that the most highly upregulated genes during both MP-12 and ZH548 infection were ISGs (interferon stimulated genes). The two most highly upregulated genes at 18hpi were interferon stimulated genes (ISGs) IFIT2 and OASL, and in our study at 48hpi those were also the top upregulated transcripts. Additional ISGs upregulated at 48hpi include the other 2’-5’ oligoadenylate synthase (OAS) family members OAS1 and OAS2, IFI44, MX2, IFNB1 and IFIH1/MDA5. Many inflammatory cytokines and chemokines were also activated (CXCL10, CCL5, IL16, ANGPT2, CXCL11). Tripartite motif containing 22 (TRIM22) was also highly upregulated, and has previously been shown to play an important role in antiviral immunity during Influenza A virus (IAV) infection due to its ability to target the IAV nucleoprotein for degradation [35]. Other upregulated genes were involved in cell adhesion (ITGAM) and microtubule motor activity (DNAH12), Wnt signaling (FAP), membrane transport (SLC2A5, NPC1L1, LAT2), metabolism (CPA2, PYGM) and apoptosis (XAF1).

Downregulated genes comprised genes involved in a diverse array of cellular functions. The most highly downregulated gene was MIR210HG, a microRNA that is upregulated in several cancers and is critical for regulating the hypoxic response [36, 37]. The hypoxia-induced lipid droplet associated HILPDA was also highly downregulated in our study and the study by Pinkham and colleagues. Downregulation of these factors is interesting, since RVFV infection stimulates oxidative stress [38]. In addition to its role in altering host cell transcription, virulence factor NSs is also known to interact with the mitochondria leading to redox imbalance and reactive oxygen species production, which leads to activation of NF-κB (p65) and p53 [39]. Ionotropic glutamate receptors (GRIA3, GRID2), a potassium-voltage gated channel (KCNA2), kinesin family member 12 (KIF12), and several long noncoding RNAs (RP11-214O1.2, RP11-355I22.7, RP11-250B2.3, RP11-148O21.6, AC083843.1) were also significantly downregulated.

### Gene ontology and KEGG pathway analysis

Gene ontology (GO) analysis of differentially expressed genes was performed using GOrilla, which allows the user to input a ranked list of target genes for analysis, and redundant GO terms were removed using REVIGO [21, 22]. Using GOrilla for a large list of genes, input of genes ranked by their log2 fold change values outputs GO terms more significantly enriched in highly differentially expressed genes. GO analysis of upregulated genes using a single ranked list of genes and a p-value threshold of 10^−3^ illustrates, as expected, that the immune response is highly enriched upon infection as well as cell surface receptor signaling and signal transduction (Fig 2C). The much shorter list of downregulated genes showed no significant enrichment using the same parameters.

KEGG pathway analysis using DAVID for all significantly differentially expressed genes indicated that several pathways, inclucing many pathways involved in the type I IFN response (NF-κB signaling, TNF signaling, toll-like receptor signaling, RIG-I like receptor signaling, cytosolic DNA-sensing) were transcriptionally modified post-infection (Table 2). As is the case with canonical type I IFN activation, it appears that IFN signaling activated the JAK/STAT pathway, which in turn upregulated interferon stimulated genes (ISGs). Pathway analysis also showed robust activation of the cytokine-mediated inflammatory response (cytokine-cytokine receptor interaction, chemokine signaling, NOD-like receptor signaling). Linoleic and arachidonic acid metabolism pathways were also altered, which could indicate that arachidonic acid-derived eicosanoids influenced the immune and inflammatory response during RVFV infection, as was previously discovered for respiratory viruses [40]. PI3K/AKT/mTOR signaling was also altered upon infection, consistent with prior work that showed RVFV infection attenuates Akt and downstream mTORC1 activity [41, 42]. Lastly, extracellular matrix (ECM) -receptor interaction pathways were modified during infection. Further investigation of the involvement of ECM receptor signaling during infection could yield insight into viral entry and cell to cell spread, which is an understudied area for RVFV.

**Table 2:** KEGG pathways analysis of differentially expressed genes.

KEGG Pathway	p-value
Cytokine-cytokine receptor interaction	2.80E-14
Complement and coagulation cascades	2.40E-05
Jak-STAT signaling pathway	2.70E-05
ECM-receptor interaction	2.00E-04
NF-kappa B signaling pathway	2.00E-04
Neuroactive ligand-receptor interaction	2.20E-04
Protein digestion and absorption	2.30E-04
Linoleic acid metabolism	6.90E-04
Hematopoietic cell lineage	4.10E-03
Chemokine signaling pathway	4.20E-03
Toll-like receptor signaling pathway	5.80E-03
Osteoclast differentiation	6.00E-03
TNF signaling pathway	6.40E-03
Arachidonic acid metabolism	7.10E-03
Phenylalanine metabolism	1.40E-02
Calcium signaling pathway	1.70E-02
Fat digestion and absorption	2.00E-02
NOD-like receptor signaling pathway	2.60E-02
Cytosolic DNA-sensing pathway	2.60E-02
Leukocyte transendothelial migration	2.70E-02
Phototransduction	2.90E-02
Steroid hormone biosynthesis	3.20E-02
Tyrosine metabolism	3.30E-02
PI3K-Akt signaling pathway	3.60E-02
Serotonergic synapse	3.80E-02
Transcriptional misregulation in cancer	4.20E-02
Natural killer cell mediated cytotoxicity	4.40E-02
RIG-I-like receptor signaling pathway	4.60E-02

### Transcription factor binding sites represented in differentially expressed genes

**Fig 3:**
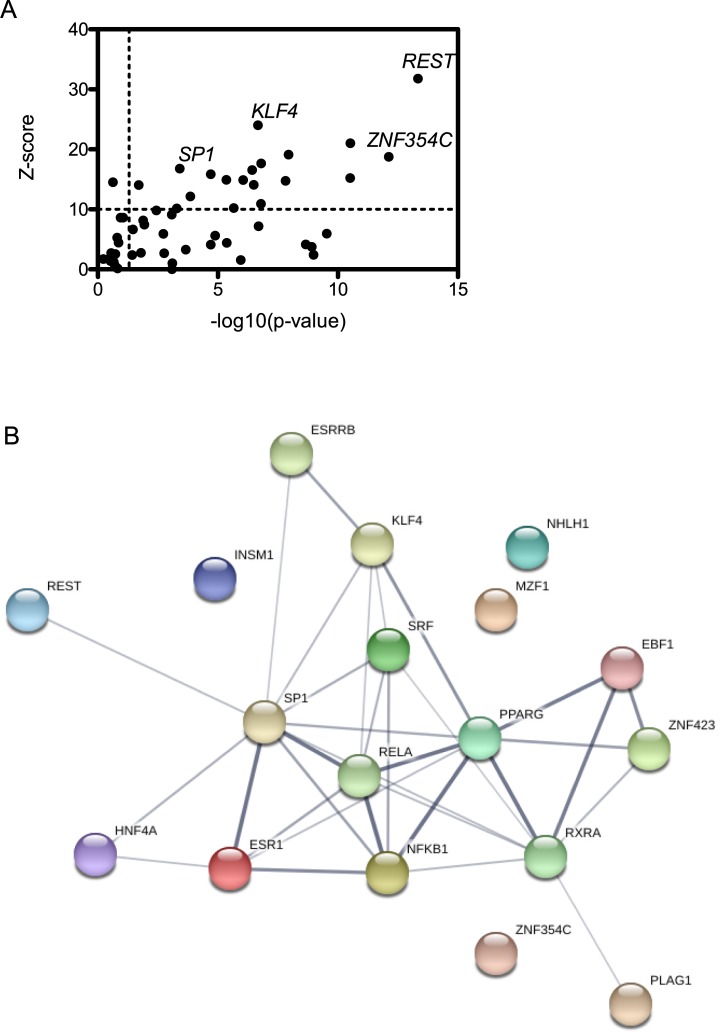
Enriched transcription factor binding sites present in differentially expressed genes. A. Statistically significant TFBS are represented in the upper-right quadrant with Z score >10 and p-value >0.05. B. STRING network analysis of statistically significant TFBS. Network edges represent interaction partners and increased line thickness at edges indicates higher confidence interactions.

As mentioned previously, the viral NSs protein is a major virulence factor that dramatically alters the host-pathogen interface during infection. Its primary biological functions include inhibition of general transcription, suppression of IFNB promotor activation, and degradation of PKR. Although NSs inhibits general transcription and specifically that occurring from IFNB promoters, it is clear that the cell can at least partially overcome NSs’s transcriptional shutdown at this timepoint post infection. In order to investigate and categorize the types of genes that may escape NSs’s block on general transcription, we looked for overrepresented transcription factor binding sites (TFBS) in the promoter regions of up- and down- regulated genes using oPOSSUM-3 software in Single-Site Analysis (SSA) mode [24] (S6 Table). SSA is used to find transcription factor binding motif enrichment in a given list of genes and assessed relative to motif enrichment in a background list of genes. Z-scores in conjunction with p-values are used to determine if a particular TFBS that occurs in the foreground set differs significantly from the expected occurrence in the background. Using a threshold Z-score of > 10 and a p-value of < 0.05, we discovered that just under 20 TFBS were significantly enriched in our DEGs (Fig 3A). In order to deduce synergistic interactions and functional networks associated with the transcription factors that interact with the identified binding sites, we performed STRING analyses where network edges represent interaction partners and thicker lines correspond to higher confidence interactions (Fig 3B). Consistent with the analysis of GO terms associated with DEGs, we found TFBS that are important for regulation of fatty acid metabolism, including PPARG, RXRA and ZNF423 [43]. Interestingly, ZNF423 also interacts with SMAD1-SMAD4 complex, which is involved in BMP and TGF-β signaling that regulates cell growth and differentiation as well as apoptosis. The SMAD family of transcription factors were also recently shown to be phosphorylated as a result of RVFV infection [44]. Further studies will be needed in order to determine whether synergy exists between ZNF423 and activated SMADs that influences RVFV infection.

A high confidence axis represented in the STRING network is the canonical NF-κB transcription factors RELA (p65) and NFKB1 (p50), which form heterodimeric complexes that act as transcriptional activators important for innate immunity, inflammation, apoptosis and cell growth and differentiation. SP1 connents to this axis, and although it is involved in a variety of biological processes and binding sites are present in many housekeeping genes, it has been implicated in cell cycle arrest and p-53 dependent apoptosis as well as in switching on early innate immune transcription [45]. ESR1 is also represented in this axis and is known to interact with NF-κB transcription factors in order to influence progression of breast cancer to advanced stage and metastatic disease [46]. However, the synergy between ESR1 and NF-κB during viral infection has not been extensively studied. During a transcriptional profiling study of Influenza A infection in lung epithelial cells, however, ESR1 was noted as a gene whose regulation was altered during NS1 deletion mutant viral infection [47]. Since the viral NS1 protein is a virulence factor responsible for repressing the host antiviral response in several ways, this study along with our work may indicate that ESR1 could play an important and underappreciated role in host activation or regulation of the antiviral response. ESR1 expression fluctuates during pregnancy, meaning that ESR1 has the potential to be uniquely important during RVFV infection, which is associated with high levels of abortion and neonatal death in pregnant ruminants [48].

### Changes in host alternative splicing during viral infection

**Fig 4:**
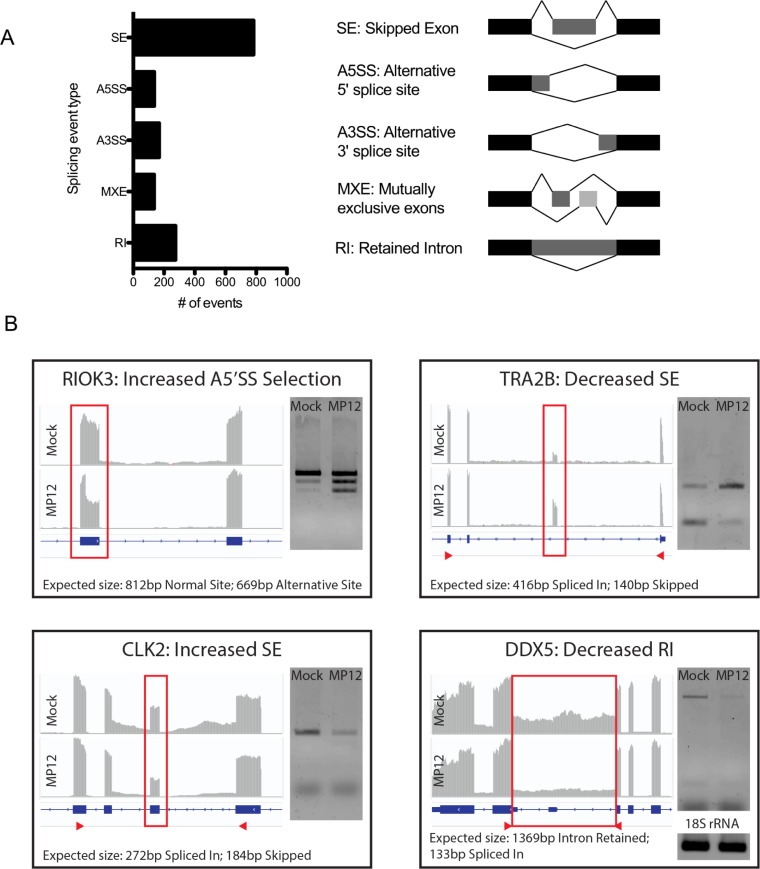
Alternative splicing events induced by viral infection. A. Significant AS events in infected compared with mock-infected cells. Splicing pattern types identified by the MISO algorithm are shown. B. Verification of AS events using RT-PCR with PCR primers flanking the spliced region. IGV was used to visualize mRNA-seq transcripts and the vertical axis represents the quantity of mapped reads for each condition. Red arrows indicate the primer amplicon region, except for RIOK3, where a larger region was amplified between exons 5 and 10.

Alternative splicing (AS) of pre-mRNAs is a critical mechanism for regulating gene expression and expanding proteome diversity. Recent studies indicate that viral infection can cause marked changes in host splicing patterns, which results from the altered post-transcriptional program undertaken by the host as a response to invasion, but also many viruses have evolved methods to interfere with and modulate host splicing [49–51]. To determine whether AS might play a role in RVFV pathogenesis, we examined differences in splicing patterns between MP-12 infected and uninfected cells using a program called MISO (Mixture of Isoforms), which estimates differential spliced isoform expression and confidence intervals using Ψ values and Bayes factors, respectively [25] (S7 Table). Analysis yielded 1499 differential AS events between infected and uninfected cells using threshold Ψ of >0.2 and BF >10. MISO categorizes isoforms as being one of five different splicing event types: alternative 5’ splice site selection (A5SS), alternative 3’ splice site selection (A3SS), mutually exclusive exons (MXE), retainied introns (RI), and skipped exons (SE). The analyses yielded SE events as the dominant splicing pattern at 783 events, which is just over half the total number of events (Fig 4A). Additionally, analyses revealed 275 RI, 168 A3SS, 137 A5SS and 136 MXE events (Fig 4A). While most genes had only one event per gene (n = 979), there were many genes that had multiple AS events per gene. These include GUSBP11 with 7 events, EEF1D and TRA2A with 6 events, WASH7P with 5 events, and 216 genes with 4, 3 or 2 events.

Several splicing events were validated via RT-PCR using primers flanking the splicing events of interest (Fig 4B). These events also produced interesting changes in the mRNA transcripts that would result in intriguing functional outcomes. For example, Rio kinase 3 (RIOK3) undergoes alternative 5’ splice site selection in exon 8 in infected cells, which generates a frameshift and introduces a premature termination codon in exon 9. This truncated RIOK3 transcript could be a target for nonsense mediated decay, or, if it were translated, it would be predicted to produce a C-terminally truncated protein product that lacks almost the entire kinase domain. Interestingly, RIOK3 has been implicated as an important player in the host antiviral response and thus this splicing event could have important regulatory consequences for these pathways post-infection [52, 53].

Transformer 2 beta homolog (TRA2B) exhibits decreased exon 2 skipping in infected cells. This demonstrates incidence of an autoregulatory splicing event that has been previously characterized. TRA2B is an SR-like protein responsible for altering splice site selection in a variety of transcripts in a concentration dependent manner. As is the case with most splicing factors, their expression must be tightly regulated, as misregulation is associated with various disorders. TRA2B uses a negative feedback autoregulatory mechanism to activate inclusion of its own exon 2. Inclusion of exon 2 generates the TRA2B4 splice variant, which is not translated into protein [54]. In infected cells, the decreased incidence of exon 2 skipping seems to indicate production of the non-functional TRA2B4 variant, perhaps the result of high TRA2B protein expression regulating its own splicing and downstream expression.

Similar to TRA2B, CDC like kinase 2 (CLK2) also autoregulates its protein expression by promoting skipping of its own exon 4. This event generates a frameshift which renders the CLK2 mRNA transcript a target of the nonsense-mediated decay machinery and is thus not translated into functional protein [55, 56]. In infected cells there is an increase in exon 4 skipping, which appears to indicate that these CLK2 transcripts are not translated. Lastly we validated the decrease in intron retention noted during viral infection in DEAD-box helicase 5 (DDX5) between exons 11 and 12. Interestingly, this intron is highly conserved and variably included in DDX5 transcripts across tissues, indicating an important functional role [57, 58]. Indeed the intron has been shown to encode a processed miRNA that coimmunoprecipitates with Ago2, although endogenous targets have yet to be characterized [59].

**Fig 5:**
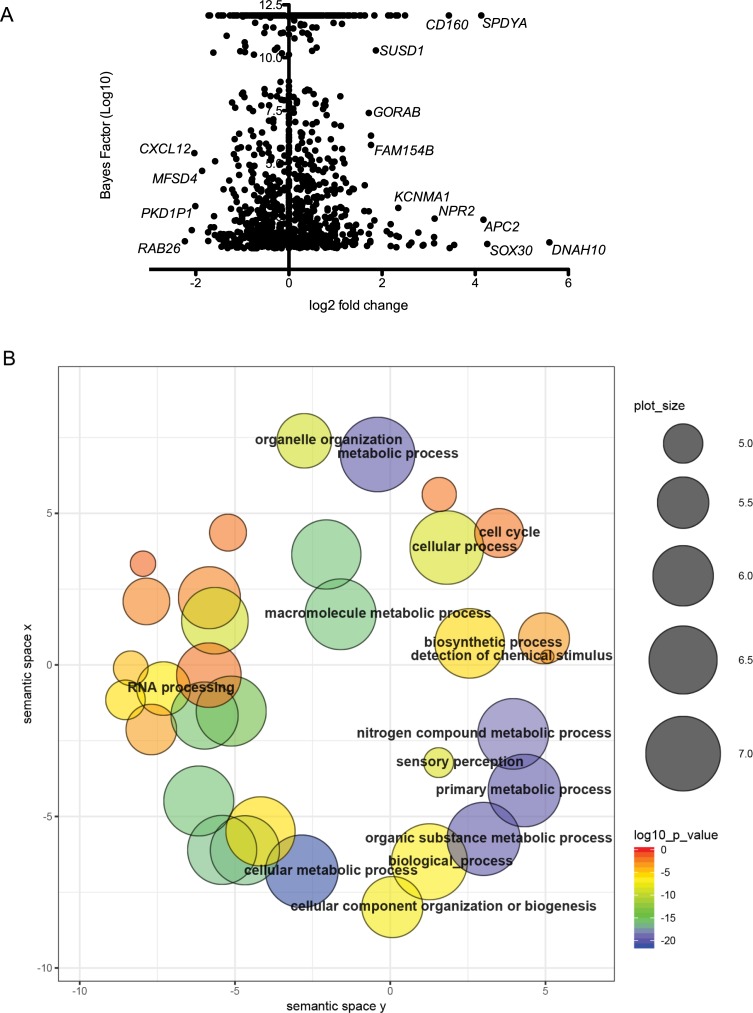
Expression and GO analysis of alternatively spliced transcripts. A. Alternative splicing events plotted with Bayes factor represented on the y-axis and differential expression on the x-axis. B. REVIGO scatterplot analysis of overrepresented GO terms associated with AS genes. The two-dimensional plot clusters GO terms based on semantic similarities. Bubble color corresponds to the p-value; size indicates the frequency of the GO term within the database.

Most of these alternatively spliced genes were not differentially expressed at the level of transcription, indicating that differential expression and AS are independent regulatory mechanisms occurring post-infection (Fig 5A). To understand the functional consequences of these splicing events, we implemented GO enrichment analysis using all AS genes. GO terms with p-values of <0.05 were subjected to Revigo in order to cluster and delete redundant GO terms. REVIGO scatterplot analysis shows that most AS genes are involved in cellular metabolic and biosynthetic processes, as well as RNA processing (Fig 5B). KEGG pathways significantly enriched amongst AS genes were mRNA surveillance pathway, Fanconi Anemia pathway, Hippo signaling, and RNA transport.

## Discussion

This study has detailed the transcriptional modifications and alternative splicing events that occur in RVFV MP-12 infected HEK293 cells. We demonstrate that many genes are differentially expressed and we identify GO and KEGG pathways as well as transcription factor binding sites in DEGs. One of the main themes that appeared in this analysis was activation of innate immunity and inflammation. ISGs comprised a majority of top up-regulated genes, and we confirmed an increase in several of these genes using RT-qPCR. In addition, KEGG pathways associated with DEGs included NF-kB signaling, TNF signaling, toll-like receptor signaling, RIG-I like receptor signaling, cytosolic DNA-sensing, JAK-STAT signaling, cytokine-cytokine receptor interaction, chemokine signaling, NOD-like receptor signaling.

Several studies have highlighted the importance of the antiviral response in determining viral pathogenesis and disease outcome as a result of RVFV infection [60, 61]. Age-associated susceptibility to RVFV has been demonstrated in humans, livestock and laboratory animals, which suggests that host resistance may be mediated by the more robust type I interferon system present in mature adults [62, 63]. Like most negative-sense RNA viruses, RVFV does not produce significant levels of dsRNA during infection [64]. Nevertheless, RVFV activates PKR, and the 5’ triphosphate groups present at the RVFV genome termini act as a strong activators of RIG-I-dependent interferon induction, culminating in ISG induction [65, 66]. Despite the fact that NSs is a powerful inhibitor of PKR signaling and IFNB promoter activation, our work shows that the host still mounts a significant antiviral response by 48hpi. Global transcriptome profiling during Schmallenberg virus infection (SBV) showed dampened activation of IFN stimulated genes in SBV infection as opposed to infection with SBV lacking the NSs gene [15]. However, similar to the results shown here, their work also indicated that some antiviral genes escape NSs suppression during SBV infection.

It has been previously shown that NSs acts as a global inhibitor of transcription via its interaction with TFIIH complex components and by binding with SAP30 to prevent IFNB promoter activation [67]. We observed only a modest decrease in total reads obtained from MP-12 infected cells when compared with mock-infected cells, and most genes in the DEG analysis were actually upregulated during infection. A similar trend was observed by Pinkham and coworkers during mRNA-seq analysis comparing MP-12 and ZH548 strains of RVFV in HSEACs at timepoints post-infection, where it was noted that, although the differences were not statistically significant, fewer reads were obtained during later times post-infection, and DEG analysis at 18hpi yielded more upregulated than downregulated genes [14]. Their study did, however, support the role of NSs as a general inhibitor of transcription, since RT-qPCR analysis of several housekeeping genes at 9 and 18 hpi exhibited decreased expression relative to mock infected cells. Chromatin immunoprecipitation with promoter sequence microarray analysis of NSs showed that several promoter regions interacted with NSs and that the interaction corresponds to decreased expression of the corresponding genes, with the notable exception of genes associated with the coagulation cascade, which actually exhibited increased expression as a function of NSs binding [68]. Interestingly, KEGG analysis in our study indicated alteration in the complement and coagulation pathway at 48hpi, possibly corroborating the ability of NSs to stimulate transcription of some gene categories. While certain families of genes were overrepresented among NSs interaction regions in the ChIP study, NSs only specifically interacted with about 10% of promoter regions represented in the array [68]. It is possible that at later timepoints post-infection, host antiviral response gene overexpression masks the transcriptional inhibition ability of NSs which may be more effective at earlier timepoints post-infection. Furthermore, mRNA-seq analysis at 48hpi represents a single snapshot in time, not providing any information to distinguish between newly transcribed mRNAs and mRNAs that are still present due to a longer half-life or regulation by mRNA decay mechanisms. A detailed study with multiple timepoints post-infection, as well as incorporating an infection scheme using an NSs-deletion mutant virus could aid in our understanding of the role of NSs in transcription.

Another prevalent theme across our analyses were altered pathways associated with linoleic and arachidonic acid metabolism, which appeared in KEGG analysis of the DEGs. Also, TFBS overrepresented in the DEGs belonged to TFs known to regulate fatty acid metabolism. The linoleic and arachidonic acid metabolic pathways are connected, as non-essential arachidonic acid can be obtained from diet or by desaturation and elongation of the essential fatty acid linoleic acid. Free arachidonic acid is an important lipid second messenger involved in cellular signaling and arachidonic acid-derived eicosanoids are critical for immune function. In particular, eicosanoids cooperate with cytokines and chemokines to influence inflammation [69]. For this reason, eicosanoids have been shown to be critical contributors to viral pathogenesis during respiratory virus infections, where severe virus-induced inflammation can determine the outcome of infection [40]. Work performed in patients infected with the hemorrhagic fever virus severe fever with thrombocytopenia syndrome virus (SFTSV), which is closely related to RVFV, has suggested that disease progression and severity are linked to inflammatory cytokine and chemokine production imbalance [70]. Eicosanoids are being investigated as potential antiviral therapies for respiratory viruses, and our work indicates that their role during RVFV infection may warrant exploration [40, 71].

Our analysis also showed that a surprising array of mRNA transcripts are alternatively spliced during infection. Preliminary analysis and validation of several events indicated that these are programmed events that have been previously characterized and have functional consequences for gene expression, as opposed to abberant events that could be construed as a byproduct of cellular disarray due to infection. Alternative splicing events occurring during infection were primarily in genes with GO terms related to RNA processing, and this was evident in the validated splicing events. Notably, DDX5, TRA2B and CLK2 all play important roles in RNA splicing and gene expression [54, 55, 72–74]. For these three genes, the alternative splicing patterns adopted during infection yielded transcripts that have been previously shown to be non-functional. Alternative splicing is appreciated widely as a mechanism for expanding genetic economy and proteome diversity. However, the coupling of alternative splicing with RNA decay pathways is also an important mechanism by which the cell can post-transcriptionally control gene expression. Although not extensively studied to this point, it is possible that this is an important mechanism adopted by the cell to fine-tune gene expression in the wake of viral infection. It is also important to acknowledge that viruses have adopted multifarious methods to thwart or hijack host RNA transcription, splicing, processing and decay [75–79]. Yet, a role for RVFV in interference with host splicing has not yet been described. As more transcriptome profiling studies are conducted during viral infection, it will be of great interest to examine the global splicing environment in order to better understand the host and viral mechanisms at play.

In conclusion, this study provides a transcriptomic perspective of RVFV infected HEK293 cells after two days of infection. Despite the powerful ability of NSs to derail the host immune response via inhibition of the IFNB promoter as well as NSs-driven PKR degradation, we have illustrated that the cell still mounts a robust antiviral response at this late timepoint post-infection. We further illustrate that functionally related groups of transcription factor binding sites were represented in DEGs, highlighting specific cellular priorities at this point during infection. Future transcriptome profiling studies incorporating an infection scheme with an NSs deletion MP-12 strain would better discern the effects of NSs. In addition, this is the first study to characterize significant global splicing changes that occur in RVFV infected cells, and we demonstrate that some of these splicing changes lead to non-productive trancripts. This emphasizes the importance of future studies addressing the functional consequences of the splicing overhaul that occurs as a result of infection.

## Supporting information

S1 Table Data quality summary. (XLSX)

S2 Table Mapping statistics. (XLSX)

S3 Table Gene expression FPKM values for all genes. (XLSX)

S4 Table Differential expression: Downregulated genes. (XLSX)

S5 Table Differential expression: Upregulated genes. (XLSX)

S6 Table Transcription factor binding sites in DEGs. (XLSX)

S7 Table MISO events. (XLSX)

S1 Fig Original gel: validation of alternative splicing events found in MISO. (TIF)

S2 Fig Validation of GAPDH as an internal control gene for relative RT-qPCR. (TIFF)
