# Supplementary figures and images for: Transcriptome profiling in Rift Valley fever virus infected cells reveals modified transcriptional and alternative splicing programs
Source: PLoS One. 2019 May 28;14(5):e0217497. doi: 10.1371/journal.pone.0217497 (PMC6538246; doi:10.1371/journal.pone.0217497)

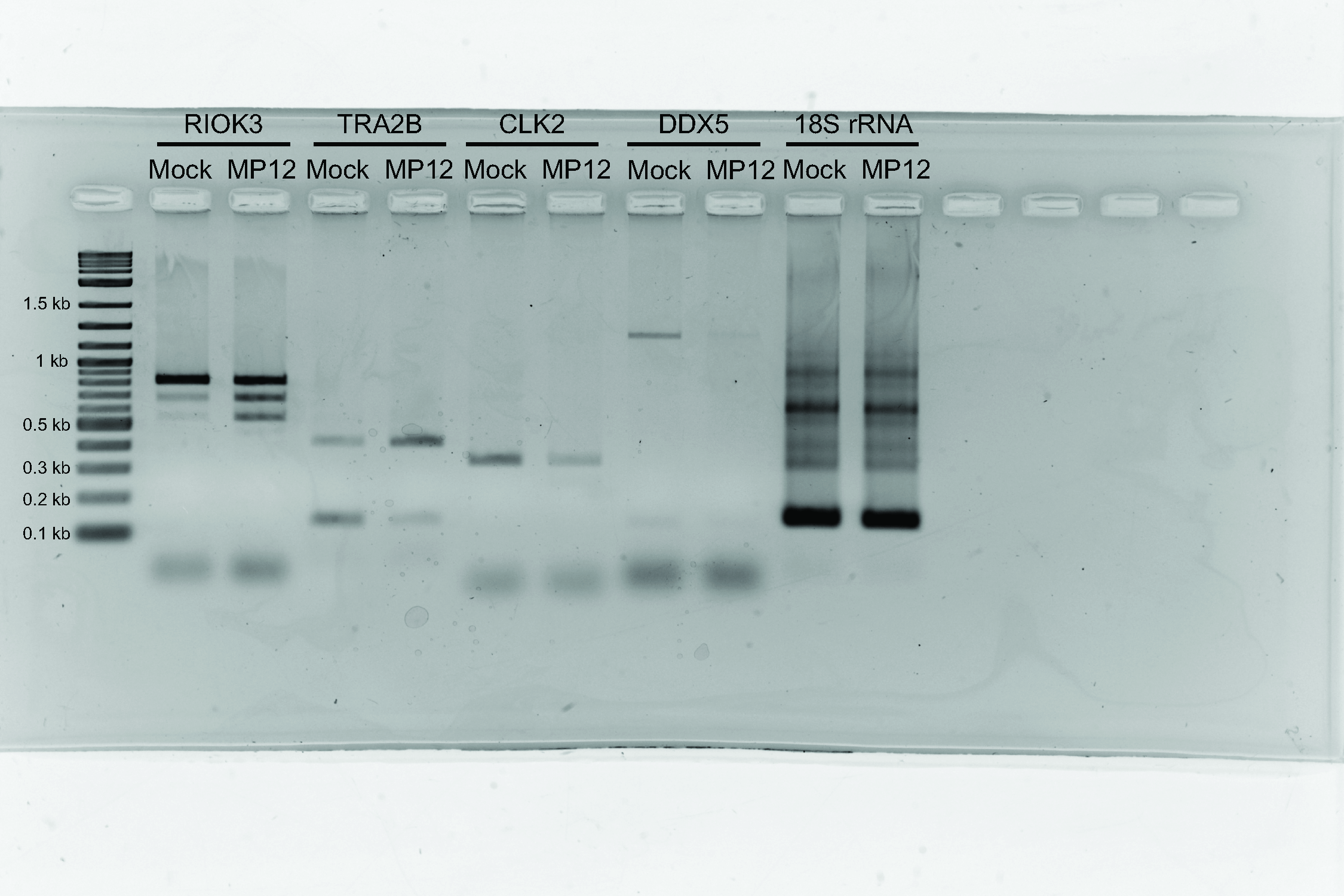

Supplement: S1 Fig — (TIF) [file pone.0217497.s008.tif]

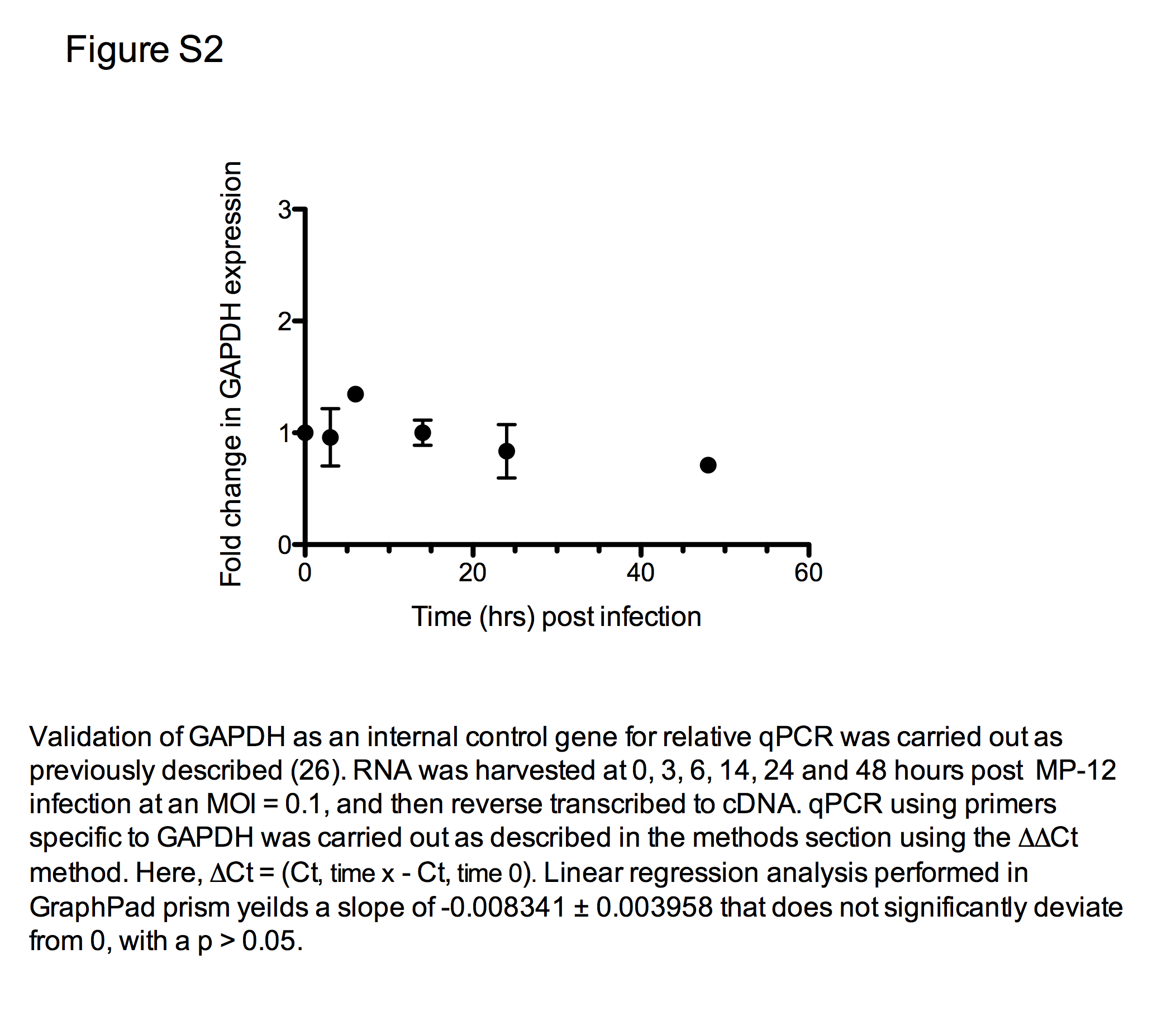

Supplement: S2 Fig — (TIFF) [file pone.0217497.s009.tiff]
